# Supplementary figures and images for: Global Distribution of Aedes aegypti and Aedes albopictus in a Climate Change Scenario of Regional Rivalry
Source: Insects. 2023 Jan 3;14(1):49. doi: 10.3390/insects14010049 (PMC9860750; doi:10.3390/insects14010049)

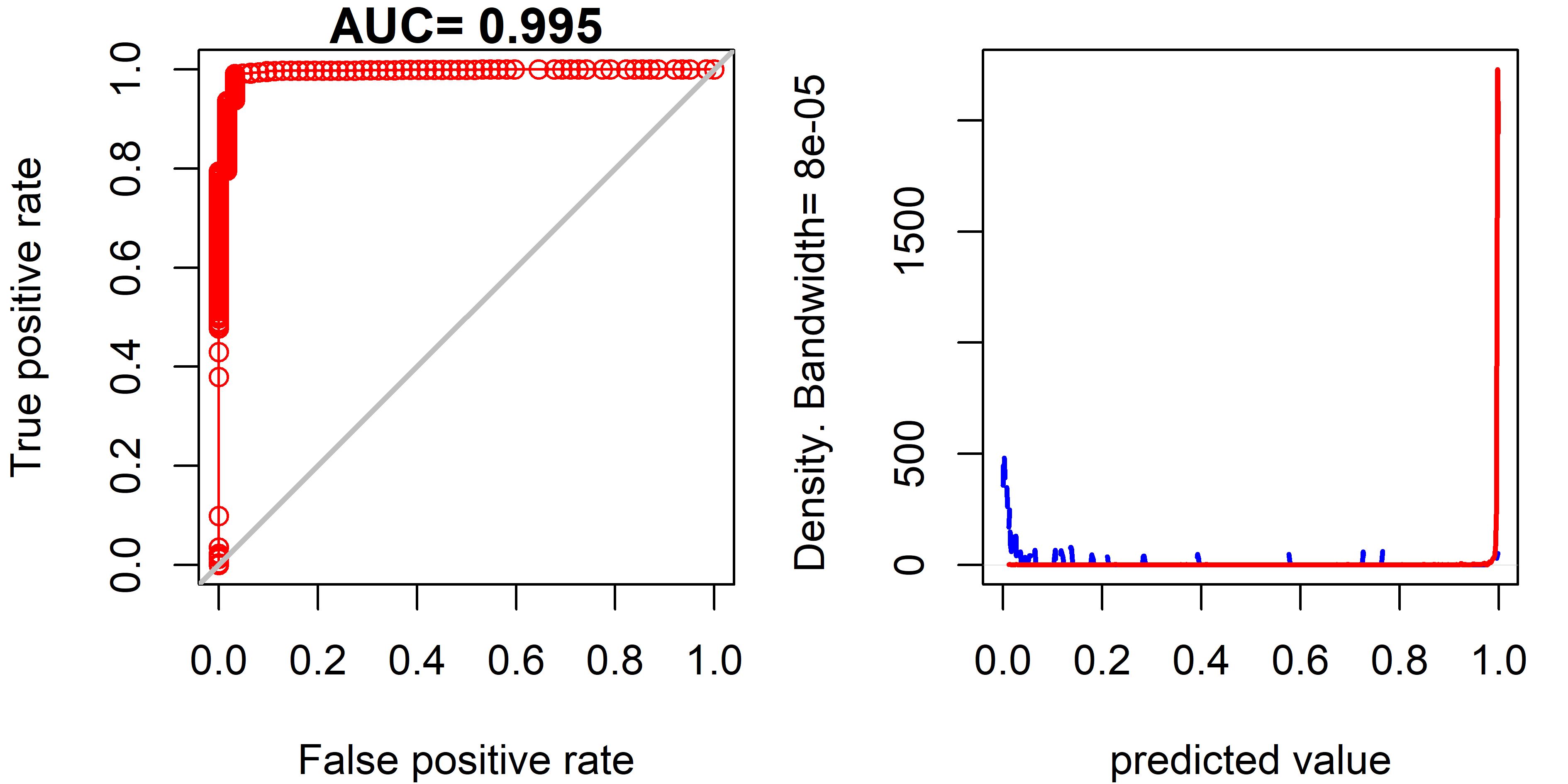

Supplement: Supplementary file 1 [file insects-14-00049-s001.zip › FigSupp1.jpg]

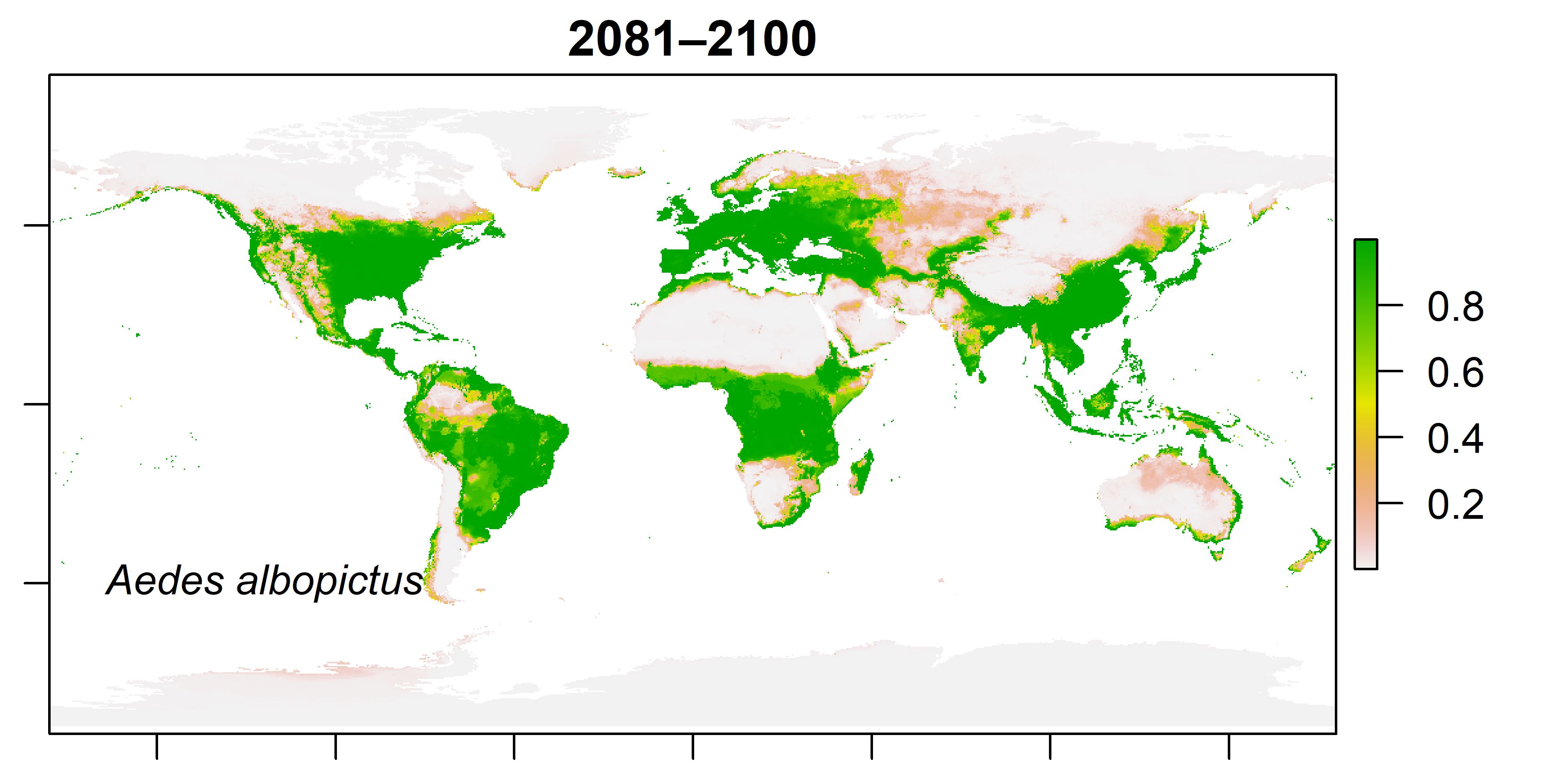

Supplement: Supplementary file 1 [file insects-14-00049-s001.zip › FigSupp10.jpg]

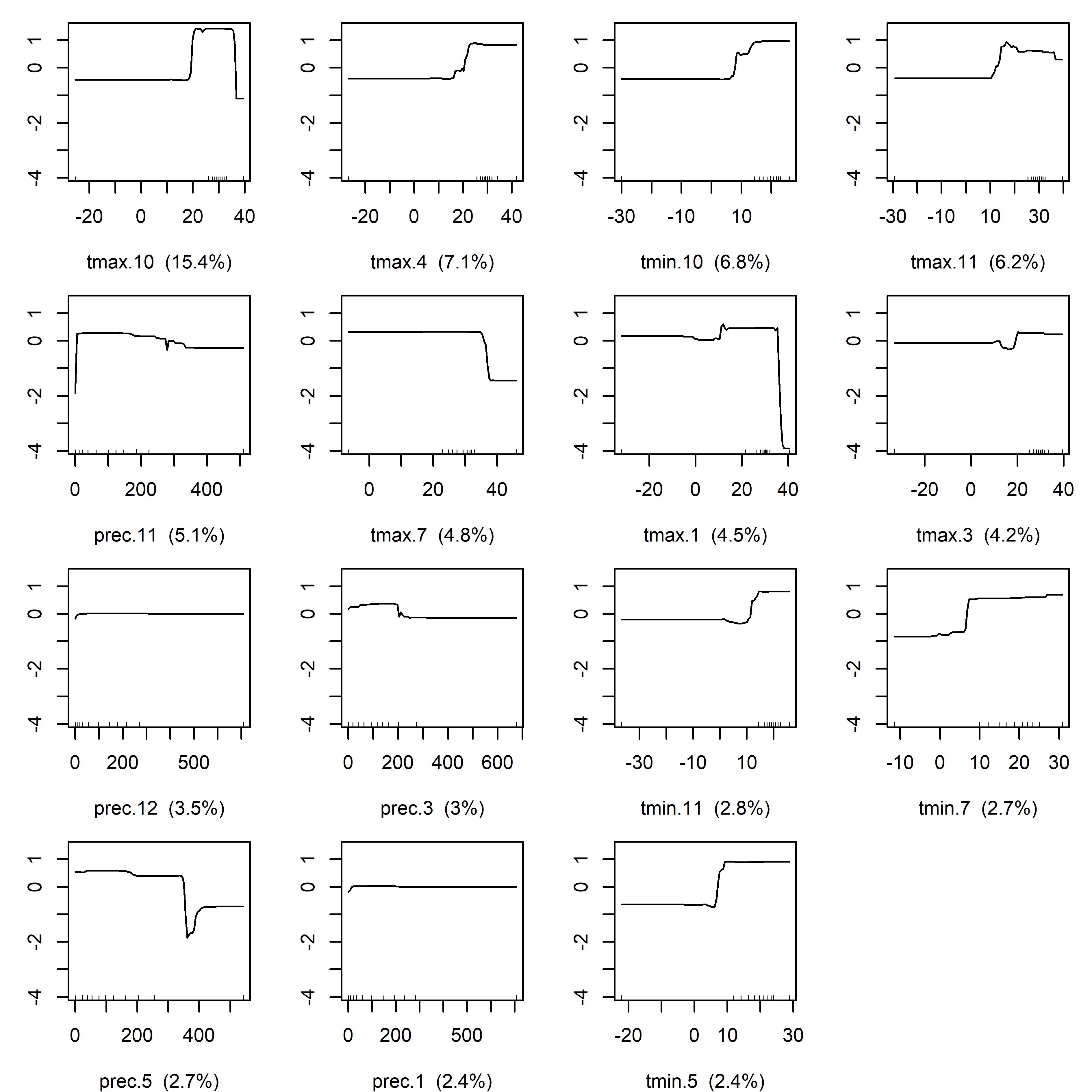

Supplement: Supplementary file 1 [file insects-14-00049-s001.zip › FigSupp2.jpg]

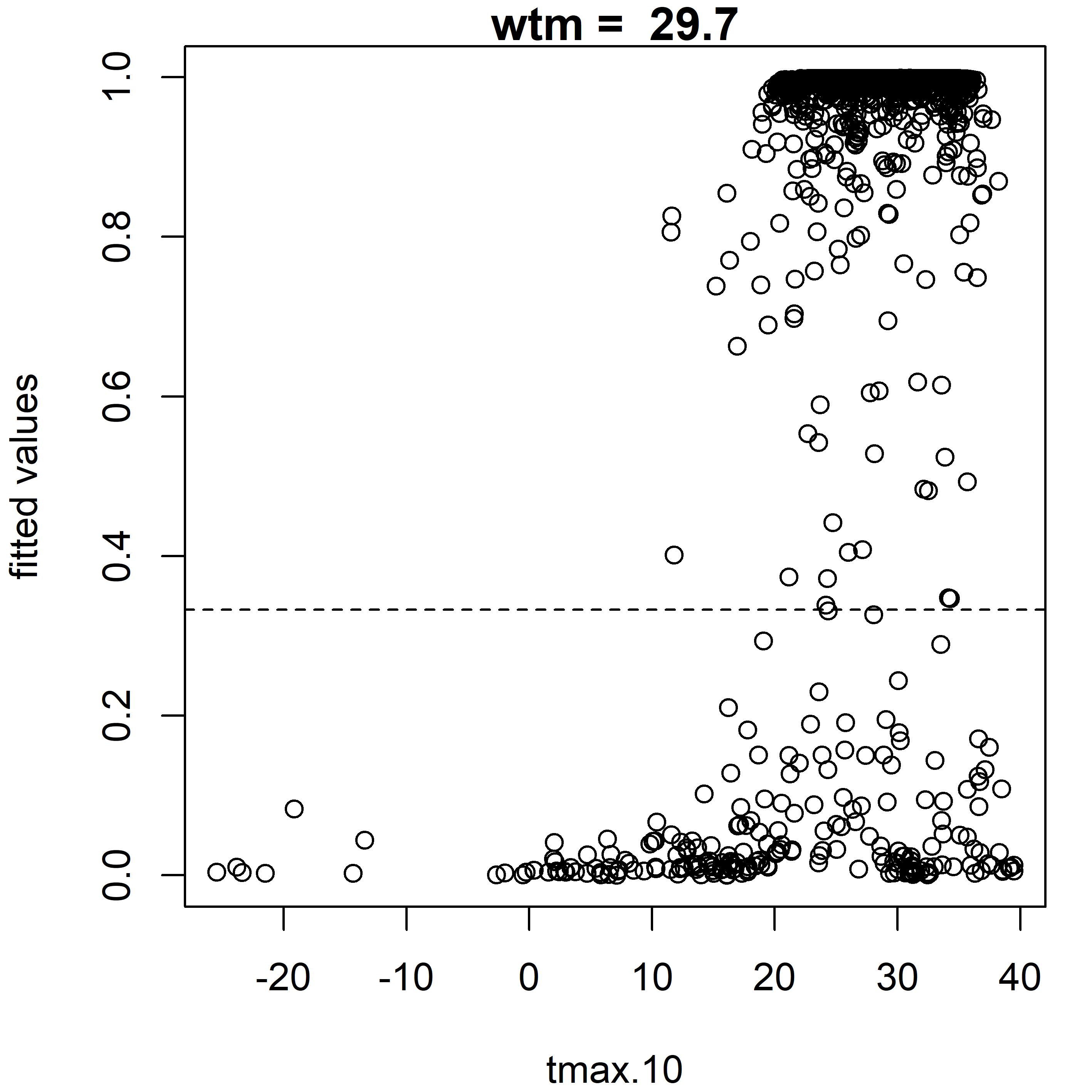

Supplement: Supplementary file 1 [file insects-14-00049-s001.zip › FigSupp3.jpg]

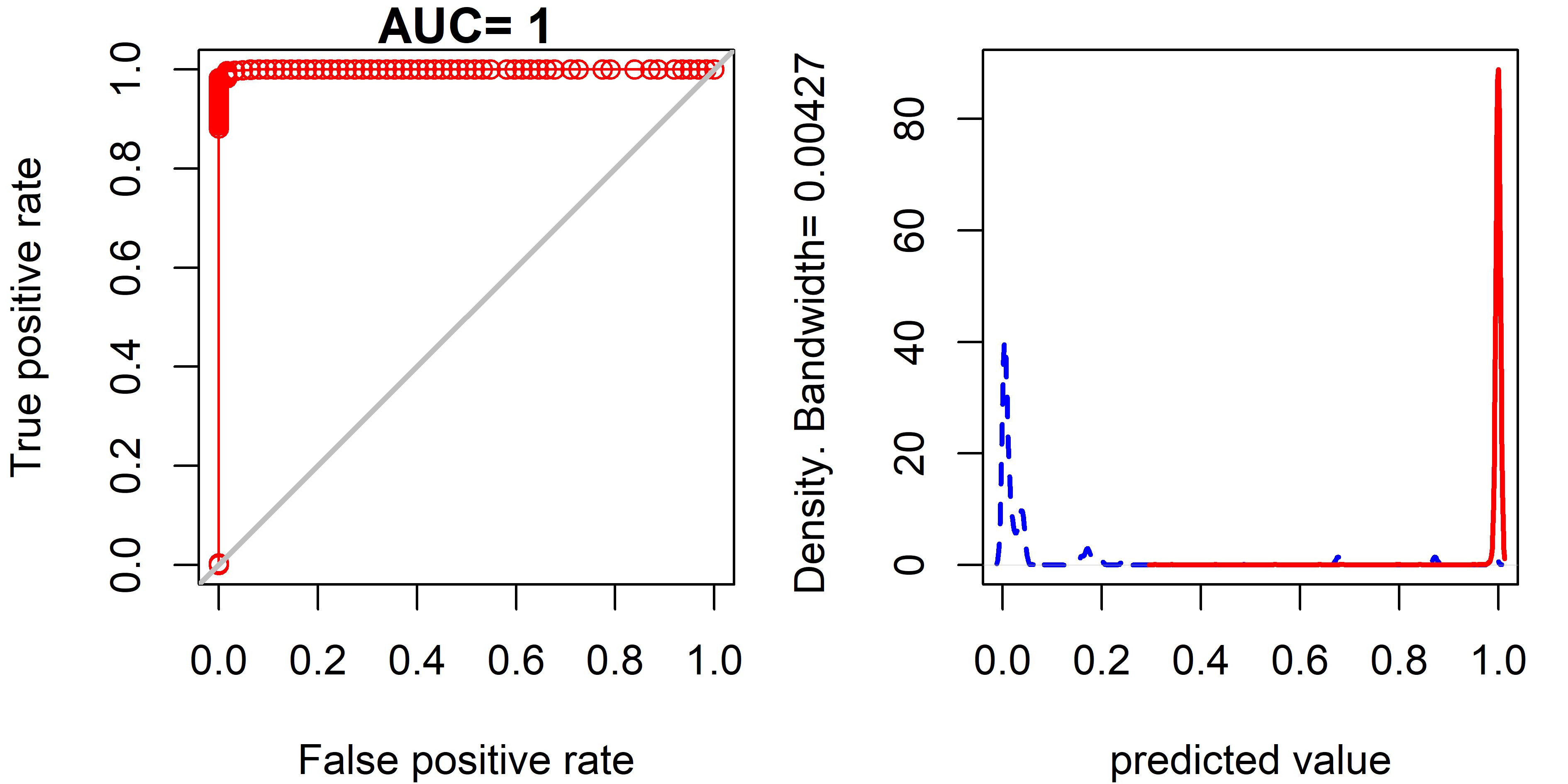

Supplement: Supplementary file 1 [file insects-14-00049-s001.zip › FigSupp4.jpg]

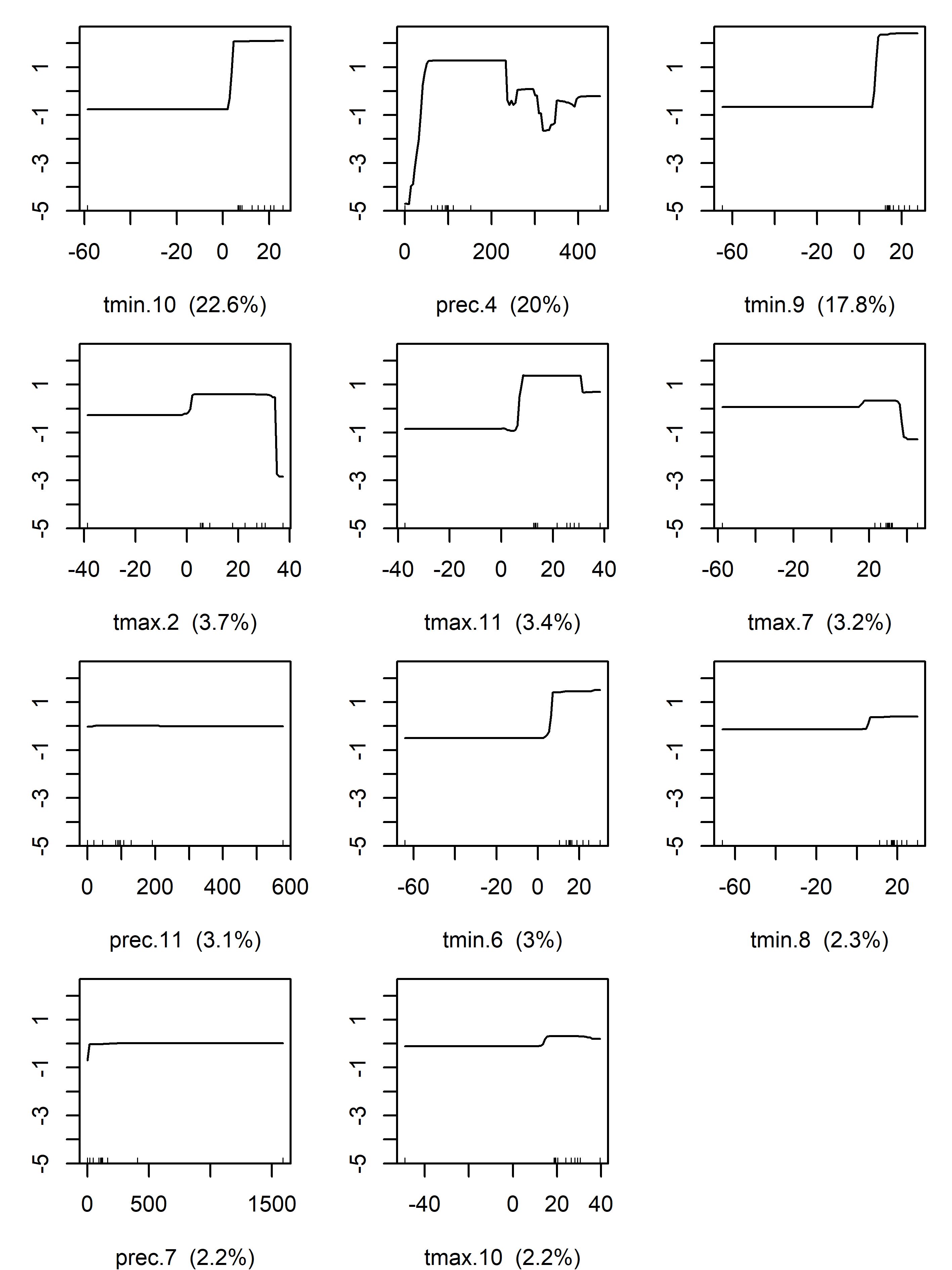

Supplement: Supplementary file 1 [file insects-14-00049-s001.zip › FigSupp5.jpg]

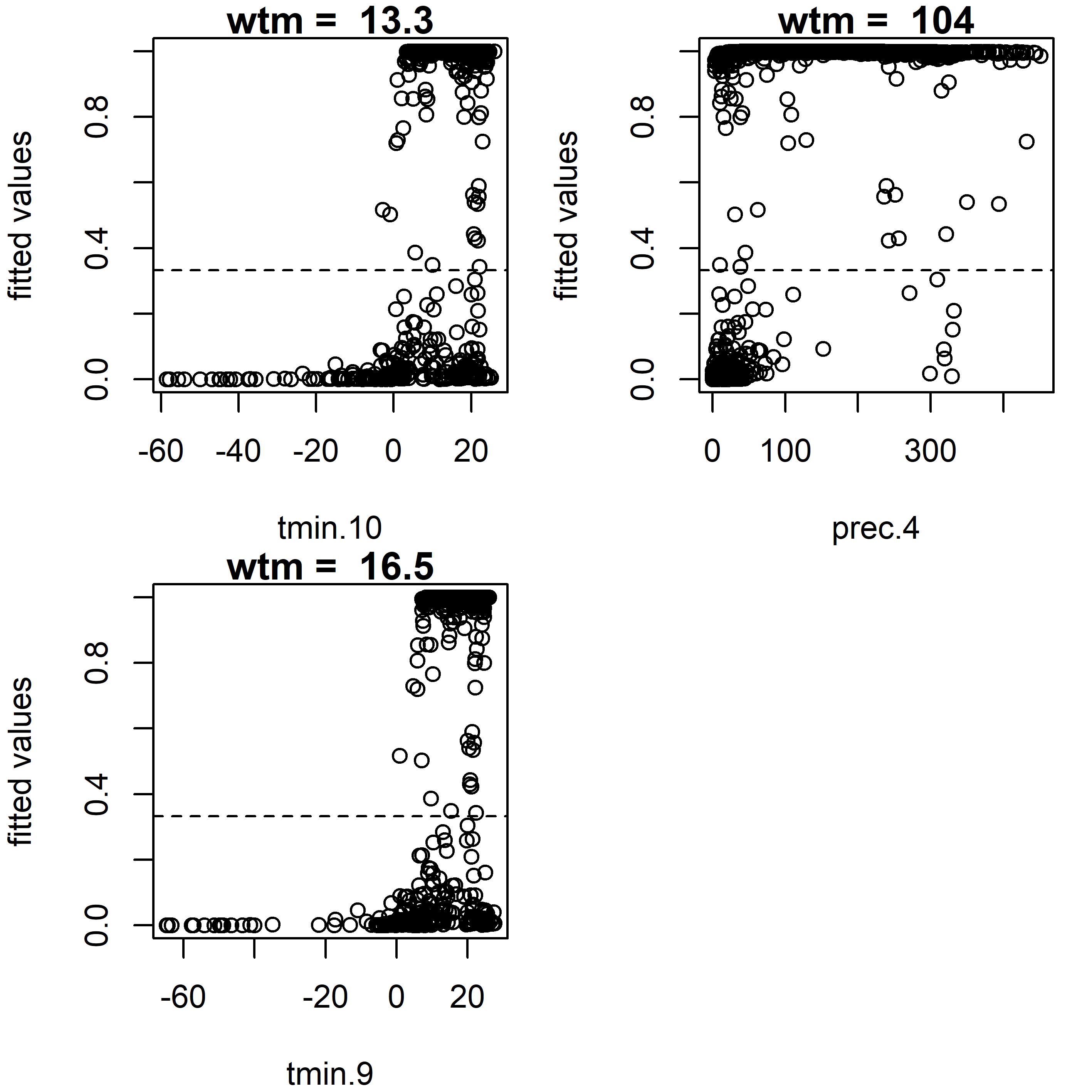

Supplement: Supplementary file 1 [file insects-14-00049-s001.zip › FigSupp6.jpg]

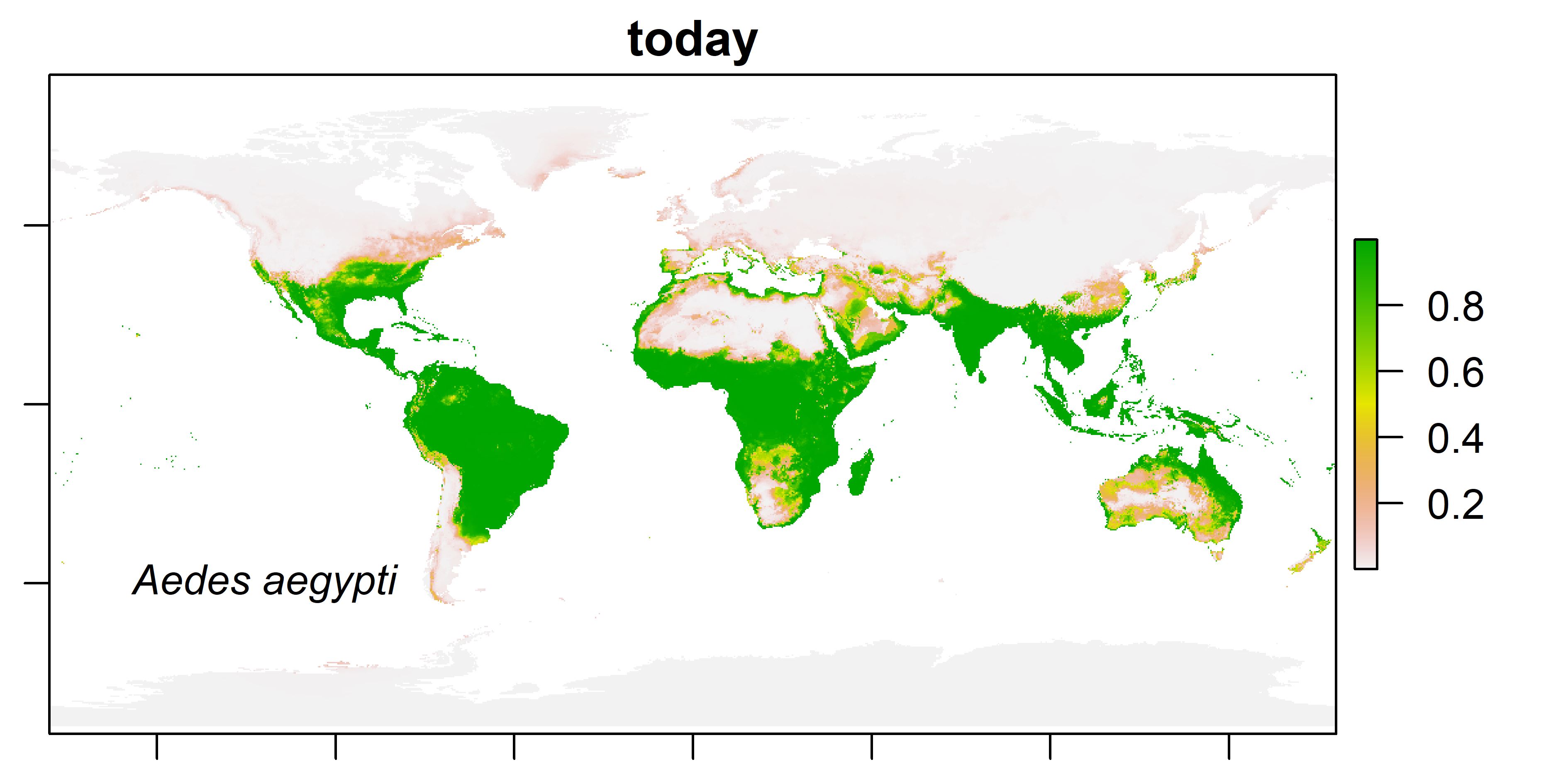

Supplement: Supplementary file 1 [file insects-14-00049-s001.zip › FigSupp7.jpg]

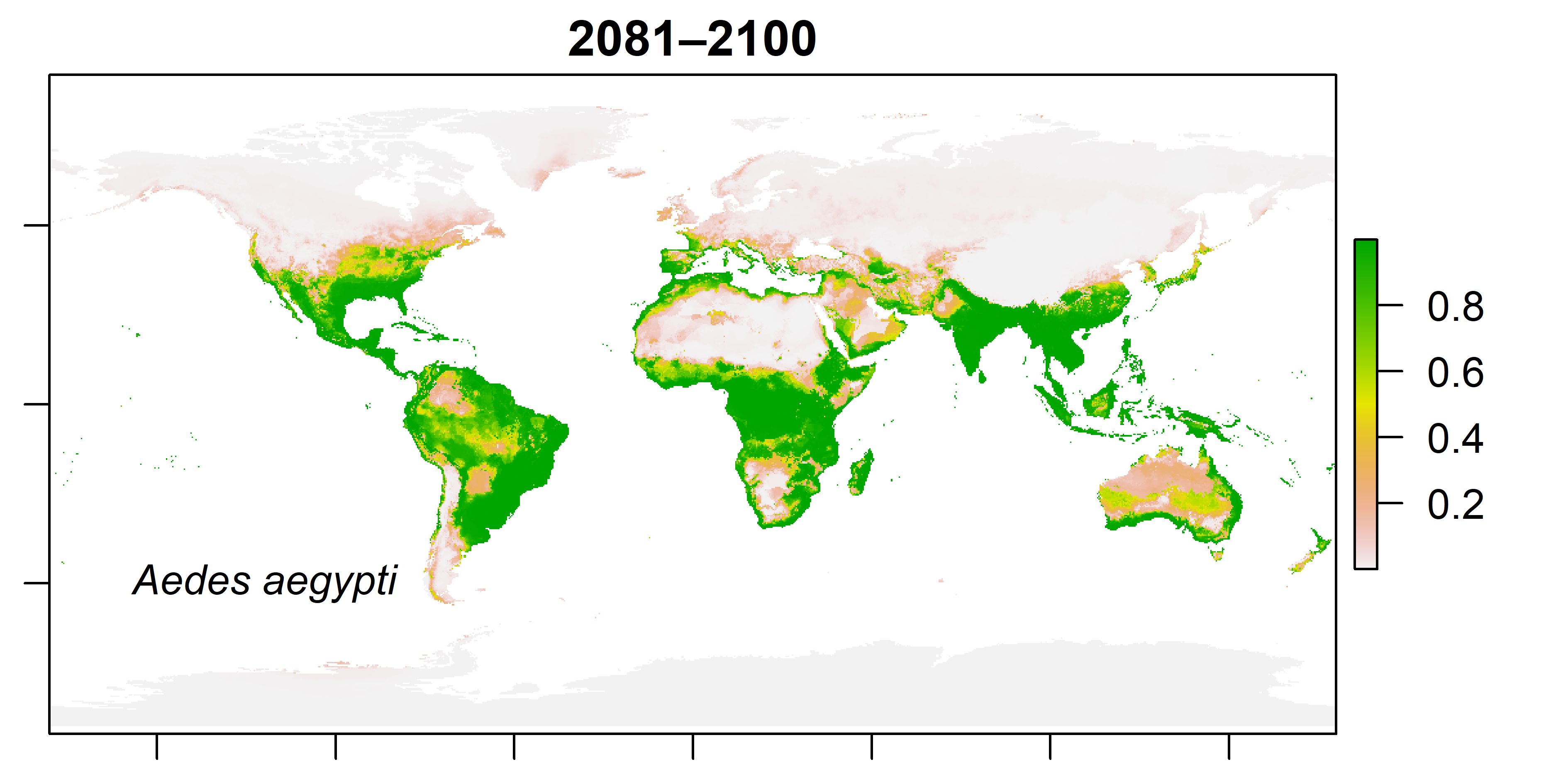

Supplement: Supplementary file 1 [file insects-14-00049-s001.zip › FigSupp8.jpg]

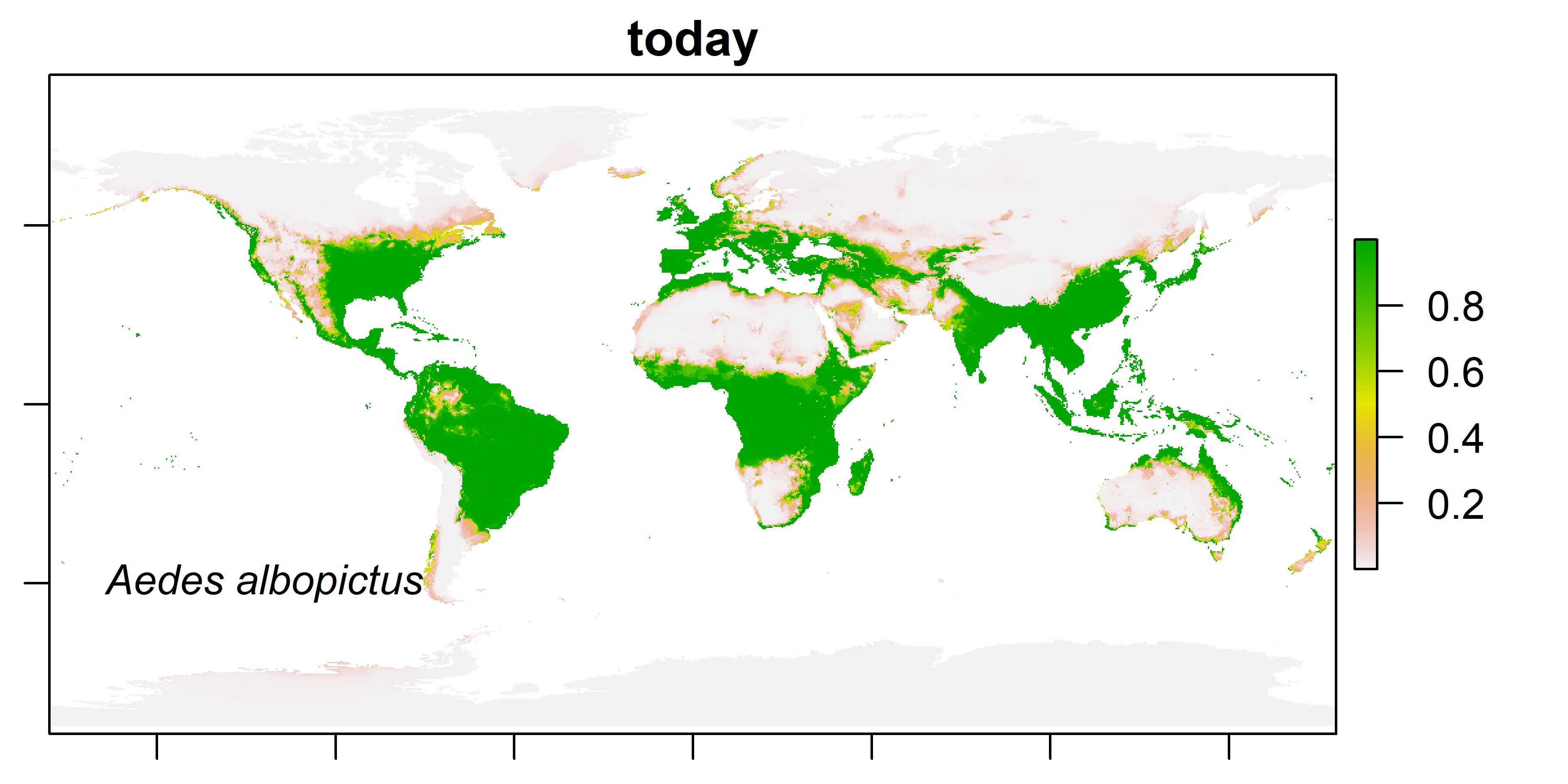

Supplement: Supplementary file 1 [file insects-14-00049-s001.zip › FigSupp9.jpg]
